# Offering vegetables to children at breakfast time in nursery and kindergarten settings: the Veggie Brek feasibility and acceptability cluster randomised controlled trial

**DOI:** 10.1186/s12966-023-01443-z

**Published:** 2023-03-28

**Authors:** Chris J. McLeod, Emma Haycraft, Amanda J. Daley

**Affiliations:** 1 Centre for Lifestyle Medicine and Behaviour (CLiMB), School of Sport, Exercise and Health Sciences, Loughborough University, Loughborough, Leicestershire, LE11 3TU UK; 2 School of Sport, Exercise and Health Sciences, Loughborough University, Loughborough, Leicestershire, LE11 3TU UK

**Keywords:** Child health, Child development, Nutrition, Public health intervention, Randomised controlled trial, Nursery, Kindergarten

## Abstract

**Background:**

In many Westernised countries, children do not consume a sufficient amount of vegetables for optimal health and development. Child-feeding guidelines have been produced to address this, but often only promote offering vegetables at midday/evening meals and snack times. With guidance having limited success in increasing children’s vegetable intake at a population level, novel approaches to address this must be developed. Offering vegetables to children at breakfast time in nursery/kindergarten settings has the potential to increase children’s overall daily vegetable consumption as children typically attend nursery/kindergarten and many routinely eat breakfast there. However, the feasibility and acceptability of this intervention (Veggie Brek) to children and nursery staff has not been investigated.

**Methods:**

A feasibility and acceptability cluster randomised controlled trial (RCT) was undertaken in eight UK nurseries. All nurseries engaged in one-week baseline and follow-up phases before and after an intervention/control period. Staff in intervention nurseries offered three raw carrot batons and three cucumber sticks alongside children’s main breakfast food each day for three weeks. Control nurseries offered children their usual breakfast. Feasibility was assessed by recruitment data and nursery staff's ability to follow the trial protocol. Acceptability was assessed by children’s willingness to eat the vegetables at breakfast time. All primary outcomes were assessed against traffic-light progression criteria. Staff preference for collecting data via photographs versus using paper was also assessed. Further views about the intervention were obtained through semi-structured interviews with nursery staff.

**Results:**

The recruitment of parents/caregivers willing to provide consent for eligible children was acceptable at 67.8% (within the amber stop–go criterion) with 351 children taking part across eight nurseries. Both the feasibility and acceptability of the intervention to nursery staff and the willingness of children to consume the vegetables met the green stop–go criteria, with children eating some part of the vegetables in 62.4% (745/1194) of instances where vegetables were offered. Additionally, staff preferred reporting data using paper compared to taking photographs.

**Conclusions:**

Offering vegetables to children at breakfast time in nursery/kindergarten settings is feasible and acceptable to children and nursery staff. A full intervention evaluation should be explored via a definitive RCT.

**Trial registration:**

NCT05217550.

**Supplementary Information:**

The online version contains supplementary material available at 10.1186/s12966-023-01443-z.

## Background

It is well known that children in many Westernised countries do not eat a sufficient amounts of vegetables for optimal health and development [1] with only 18% of UK children aged 5–15 years eating five portions of fruit or vegetables per day [2], the national recommended daily intake, and one in three 5–10-year-olds eating less than one portion of vegetables per day [3]. Furthermore, a diet based on energy-dense highly-palatable foods lacking in vegetables is associated with the onset of noncommunicable diseases such as obesity, some cancers, and cardiovascular diseases [4–7]. As such, increasing children’s intake of vegetables from early in life is a public health priority.

Targeting children’s exposure to vegetables is pivotal for increasing the likelihood that they will learn to eat these frequently rejected foods [8, 9]. To support caregivers (e.g., parents, guardians, childcare providers) in optimising children’s vegetable intake, practical guidelines have been developed [10] with many countries also implementing public health strategies to promote vegetable consumption early in childhood [11]. However, even when evidence-based child-feeding guidance is implemented, the opportunities for offering vegetables to children in many Westernised countries tend to be limited to specific times of the day; these being, midday and evening meals, and snack times, with breakfast not typically considered a time for the consumption of vegetables. This is likely because of a life-long process of learning, through conditioning and reinforcement, about the association of particular foods with particular contexts [12]. For example, in the UK, many people traditionally associate porridge and cereals with breakfast, and a chicken sandwich or salad with a midday meal [13]. Food-to-context associations are especially prevalent at mealtimes with the consumption of particular foods (e.g., salads and vegetables) being incongruous with particular mealtimes (e.g., breakfast) [13]. These associations develop from as young as 2-years-old [14, 15] (and persist into adulthood [16]) as caregivers offer their children foods at certain times of day due to their implicit considerations of food-to-mealtime appropriateness, that tend to be driven by traditions and social norms [13]. It is important to note that there are no medical, nutritional or physiological reasons as to why particular foods (e.g., vegetables) should not be consumed at particular times in the day (e.g., breakfast). Indeed, in some countries (e.g., Finland, Japan, China, Romania) breakfast foods are often the same as foods at midday or evening meals [17], with vegetables frequently part of children’s typical breakfasts [18–21].

With data suggesting that current child-feeding guidelines and interventions implemented across childcare settings have had limited success at increasing children’s vegetable intake to date [3, 22], it is important to consider more innovative ways to address this public health priority. One such novel intervention to consider is offering vegetables to young children (aged 18 months to 4 years) at breakfast time alongside their main breakfast food in nursery/kindergarten settings [23]. For context, UK nurseries are universally accessible to UK families in employment and/or who receive particular government benefits and/or financial support [24, 25]. Many UK nurseries offer breakfasts to all children in their care with families choosing whether their child attends for breakfast or arrives later in the day. Guidance around breakfast provision and recipes are provided by the UK Government (e.g., [26]) and the Office for Standards in Education, Children’s Services and Skills (Ofsted) inspections of nurseries review the provision of nutritious foods.

The rationale for offering children vegetables at breakfast time in nursery/kindergarten settings is based on the premise that 1) nursery/kindergarten-age children should have limited pre-conditioned negative associations between vegetables and breakfast time, 2) children typically attend nursery/kindergarten as part of the caring and development process, therefore targeting this setting has the potential for widespread adoption, 3) children frequently consume breakfast at nursery/kindergarten, as well as vegetables as snacks, and 4) offering vegetables at breakfast is already part of current government guidance for early-years settings in countries such as England ( [27] p.11), but is seldom implemented. However, research has not assessed young children’s acceptance of eating vegetables at breakfast time in a nursery/kindergarten setting, nor the potential barriers for staff in implementing this intervention.

If offering vegetables to children at breakfast is acceptable, this should in turn increase children’s exposure to, and consumption of, vegetables at breakfast time, thereby facilitating the development of a positive association between vegetable consumption and breakfast from an early age. Importantly, it would also provide another opportunity in the day for children to consume vegetables – an important aim as greater exposure to vegetables at nursery is associated with greater vegetable intake over time [28] – increasing their total daily vegetable (and nutrient) intake, thus improving their overall health status. However, an evaluation of the feasibility and acceptability of such an intervention to young children and nursery/kindergarten staff is required before proceeding to a definitive randomised controlled trial (RCT) to test the effectiveness of such an approach.

## Methods

### Study design

**Fig. 1:**
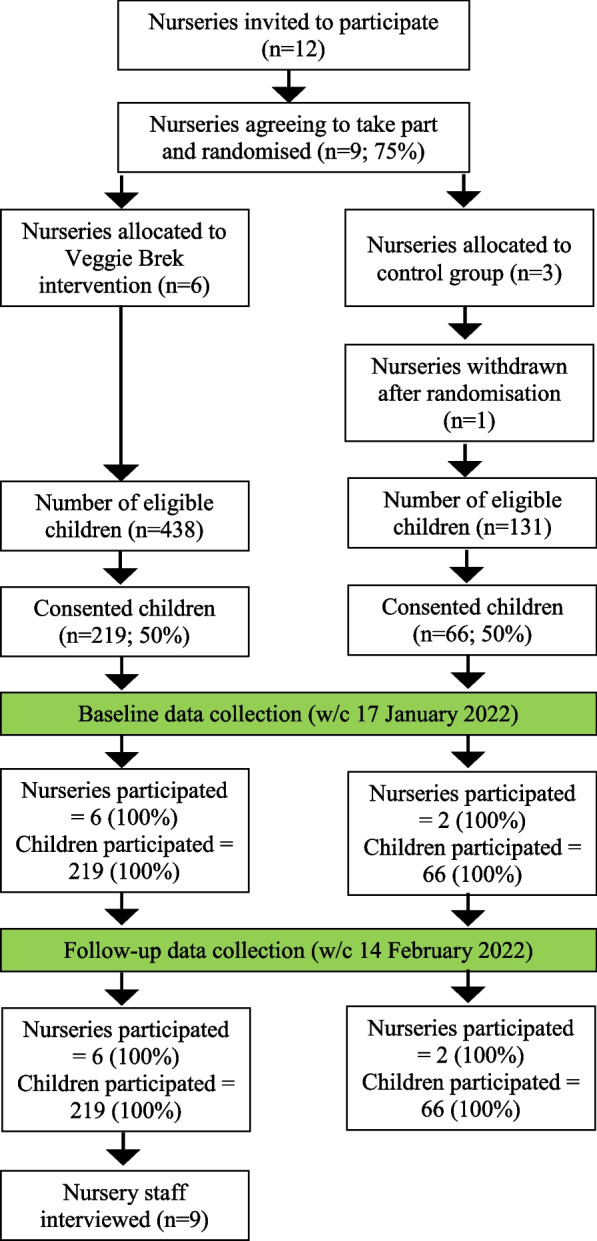
Participant flow diagram

Nurseries, nursery staff and their attending children were recruited to the Veggie Brek feasibility and acceptability cluster RCT, with embedded post-intervention semi-structured interviews conducted with nursery staff to support process evaluation of the three-week intervention. The trial schema is outlined in Table 1 and participant flow is shown in Fig. 1. This study protocol received a favourable ethical decision by the Loughborough University Ethics Review Sub-Committee (2021–5764-5234; 2022–5764-8166) and was preregistered on clinicaltrials.gov (ID: NCT05217550).

**Table 1:** Outline of the study design

	**Cluster randomised controlled trial**					**Qualitative evaluation**
	**Week 1** (17 Jan 22) **(Baseline)**	**Week 2** (24 Jan 22)	**Week 3** (31 Jan 22)	**Week 4** (7 Feb 22)	**Week 5** (14 Feb 22) **(Follow-up)**	**Weeks 6–7** (21 Feb 22—4 Mar 22)
**Intervention group**	All children served vegetables with their normal breakfast food	Intervention – all children served vegetables with their normal breakfast food			All children served vegetables with their normal breakfast food	Staff follow-up interviews
**Control group**		Control – all children served their normal breakfast food				N/A

### Co-creation, advisory group and piloting

Co-creation of the intervention protocol and piloting were undertaken at the Loughborough University Nursery (Leicestershire, UK). Researchers and advisory group members (three nursery staff members) met twice before piloting the planned intervention over two days in November 2021. Nursery staff were observed carrying out the intervention tasks and subsequently provided feedback about the protocol, resulting in minor adjustments which are reflected in the study described below. The pilot nursery and staff did not take part in the feasibility trial reported here.

### Participants, recruitment and eligibility

#### Nurseries

Nurseries of varying size (i.e., < 10 to > 200 children) and funding options for children’s attendance (i.e., local-authority and privately funded) were recruited in the East Midlands, UK, to assess the feasibility and acceptability of the intervention across a variety of nursery structures. Initial contact was made with nurseries via email, providing details about the study and inviting them to participate. All nurseries received a £50 Amazon gift card upon completion of the study to thank them for their time.

#### Children

Children were recruited if they were aged 18 months to 4 years (inclusive), engaged in complementary feeding and were able to self-feed, and ate breakfast at their nursery at least once a week. Children were excluded if they had allergies/intolerances to the intervention foods or any conditions which impacted feeding or eating. Consent for the children to participate in the study was obtained from parents/caregivers via an opt-in consent process, prior to children commencing the study. All communication with parents/caregivers (i.e., to share participant information and consent form) was facilitated via the nursery through their normal communication channels (e.g., via email, app and/or in person). Children’s demographic data (age, sex, name of nursery) was provided by parents/caregivers during the consent process.

### Randomisation

The unit of randomisation was the nursery (cluster) at a two 2:1 (intervention:control) ratio. A 2:1 randomisation was implemented to maximise the number of nurseries who experienced the intervention, while also maintaining an RCT design, to help inform a definitive trial. Randomisation occurred while nurseries were collecting consent from parents/caregivers and was conducted by an independent researcher. Nurseries were informed of their group allocation approximately two weeks prior to baseline.

### The Veggie Brek intervention

Nursery staff were asked to offer children in their nursery [room] three carrot batons and three cucumber sticks at breakfast time alongside children’s main breakfast food (e.g., cereal, toast). Nursery staff were asked to offer the vegetables to the children every weekday for three consecutive weeks. Vegetables (raw carrot batons and raw cucumber sticks) stored in plastic containers were prepared by the research team who ensured baton/stick-size uniformity (without weighing each piece) before being delivering the containers to the nursery on Mondays and Wednesdays. As the nursery staff offered breakfast to the child, they were asked to inform children that ‘there were also some carrot and cucumber pieces for them to eat too, if they wished’. Children were offered their main breakfast with the vegetables presented alongside using usual nursery crockery. All nursery staff delivering the intervention were provided with a training manual and their line manager ensured staff had read and understood the manual. The manual included detailed instructions about the trial procedures, responses to frequently asked questions and contact details to discuss any issues with the researcher.

### Control group

During the 3-week intervention phase, the control group children were offered their usual breakfast and no other intervention.

### Outcomes

The primary outcome of this study was to assess the feasibility and acceptability of the intervention according to pre-specified progression criteria (see below). Feasibility was assessed by the recruitment of children to the study and by nursery staff's ability to follow the protocol by completion of the data collection sheets. Acceptability in the intervention nurseries was determined by children’s willingness to eat the vegetables at breakfast time as assessed by the amount of vegetables eaten. These data were supplemented by post-intervention semi-structured interviews with nursery staff.

### Criteria to determine progression to definitive trial

Quantitative criteria were established with reference to a previous feasibility and acceptability cluster trial that recruited 2–4-year-olds for a healthy-eating intervention in childcare settings [29]. A traffic light stop–go criteria was implemented: ‘Green’: Proceed to definitive RCT. ‘Amber’: Proceed to definitive RCT with modifications – assess suitability of conducting study (consider in the light of other variables) and ask nursery staff for their recommendations for improving the protocol where this is required. ‘Red’: Not recommended to proceed to definitive trial.

The criteria for assessing the feasibility and acceptability of the intervention were mapped onto the study’s primary outcomes:

1. Children’s willingness to eat some part of the vegetables when offered to them at breakfast (intervention nurseries):
  Green: > 60% (of instances where vegetables were offered)
  Amber: 40–60%
  Red: < 40%
2. Number of instances where data collection by nursery staff is incomplete:
  Green: < 30%
  Amber: 30–40%
  Red: > 40%
3. Proportion of eligible children whose parents/caregivers opted them into the study:
  Green: > 80%
  Amber: 60–80%
  Red: < 60%

### Data collection

Data collection is described in Table 1. All children in both groups were served their usual breakfast plus the vegetables in week 1 (baseline). In weeks 2–4 the intervention nurseries (only) continued to serve vegetables to children at breakfast time. In week 5 all children in both groups were served vegetables at breakfast (follow-up). The purpose of the control group was to understand the willingness of nurseries to be randomised to the trial groups, to collect additional data to support the assessment of child recruitment, to test out baseline/follow-up study processes, and to mimic the design of a potential definitive RCT; no other data from the control group is reported here. In all weeks of the study, nursery staff reported on data collection sheets (see Additional File 3) whether they had offered the vegetables to each child (indicated by checking a box) – and if a child was not offered the vegetables the reason why – and the amount of each vegetable piece eaten by drawing a circle on a graphical depiction of three carrot batons and three cucumber sticks which were segmented into three equal sections. Staff did not note whether children wanted (and were offered) more vegetables beyond the three carrot batons and three cucumber sticks they were offered.

#### Using photographs to record consumption

In addition to the data collection described above, a further goal was to test the feasibility and acceptability of an alternative data collection method. Intervention group nursery staff were asked to also take photographs of the vegetables leftover on children’s plates to understand whether this approach to data collection was feasible, acceptable and preferable for nursery staff to use to report vegetable intake. Nursery staff were asked to place a label with the date, child’s name and nursery room next to the vegetables and to take a photograph of the child’s leftover vegetables (via the nursery’s electronic tablet) on the second week of the intervention phase. Nursery staff then shared the photos with the researcher at the end of that week.

### Sample size

As this was a feasibility trial to inform the design of a subsequent effectiveness trial, a formal sample-size calculation was not conducted [30]. A sample size of between 60 to 100 participants for the estimation of rates has been recommended for feasibility trials [30].

### Qualitative evaluation

Nursery staff were recruited from the intervention nurseries to take part in a 20–30-min semi-structured interview after completing the intervention, to provide their views about the intervention, their experience of offering children vegetables at breakfast time, their thoughts about children’s responses to consuming vegetables at breakfast time, and the feasibility and acceptability of delivering the intervention (see Additional File 2 for interview schedule). To take part, nursery staff had to be aged 18 years or over, have worked at their nursery for at least 3 months prior to the study start date, have supervised children at breakfast time at their nursery on ≥ 6 occasions across the trial duration, and be able to read, speak and understand English. Eligible nursery staff who agreed to participate provided written informed consent. Interviews were conducted either in person or via video/telephone. Nursery staff were provided with a £10 Amazon gift card to thank them for their time.

### Data analysis (trial)

Data are presented descriptively as this trial focussed on the feasibility and acceptability of the intervention to assess progression to a definitive RCT. Where data were missing or not clear, they were treated as missing data. Recruitment was quantitively measured by calculating the percentage of children whose parents/caregivers opted them into the study out of all eligible children (i.e., children who ate breakfast at nursery at least once a week and met all inclusion criteria).

Acceptability of the study to nursery staff was measured by the percentage of blank cells on the data collection sheets out of all cells where this data should have been recorded by nursery staff across all days/weeks of the intervention phase (i.e., missing data).

Children’s willingness to eat vegetables at breakfast was measured by the number of vegetables eaten (or parts of a vegetable) on all days/weeks of the intervention phase and this data was tabulated by coding the amount of vegetables eaten as recorded on the data collection sheets. As data collection was recorded according to segmented amount (in thirds of a vegetable), the amount of vegetables eaten was coded as having a maximum value of 9 counts per vegetable type (and a minimum of 0 where none of the vegetable was eaten). For example, if nursery staff indicated that a child ate all three carrot batons and all three cucumber batons offered to them, this would be coded as 9 counts of carrot and 9 counts of cucumber consumed. It was decided that counts of vegetable consumed would be determined at a precision of 0.5 counts (i.e., when half a segment of a vegetable baton/stick was circled by nursery staff) to ensure that the extent to which children were willing to try a vegetable was accurately recorded. This coding procedure was duplicated for the photograph data of leftover food (process outcome), with the first author determining counts of vegetables eaten directly from the photographs.

### Data analysis (semi-structured interview)

Interviews were audio recorded, transcribed and analysed via framework analysis [31], an approach which involves the creation of an a priori coding structure, via the study’s research questions and initial engagement with the data, with identified patterns in the full dataset mapped onto the coding ‘framework’ [32].

## Results

Qualitative data are presented alongside the quantitative data for each sub-section of the results (recruitment, acceptability, children's willingness to try vegetables, and use of photographs to record vegetable intake).

### Recruitment (nurseries and children)

Twelve local nurseries were invited to take part in the study. Three declined due to being too busy (*n* = 2) or not being able to make devolved decisions in a national franchise (*n* = 1), leaving nine nurseries who consented and were randomised. One nursery recruited only one child out of the 12 that were eligible and was therefore excluded from the study resulting in eight nurseries taking part. From a population of 569 eligible children aged 18 months to 4 years (inclusive) across these eight nurseries, 285 children (mean age = 33.2 months [SD = 9.3]) had opt-in consent from their parent/caregiver to participate. The control group comprised two nurseries with 66 children (females = 38, males = 28, mean age = 34.0 months [SD = 9.7]), and the intervention group comprised six nurseries with 219 children (females = 108, males = 111, mean age = 32.9 months [SD = 9.2]). Total recruitment duration (from contact with the first nursery to completing consent collection for all nurseries) was 16 weeks.

The average recruitment of children per nursery was 67.8% (range: 29.9–100.0%) (see Table 2 for recruitment per nursery), placing in the amber range of the progression criterion. The qualitative evaluation data highlighted that seven (of nine) participants reported no issues in relation to collecting consent from parents/caregivers, with the other two interview participants not involved in the recruitment process. Staff commented that they found the recruitment process “*absolutely fine*” [ppt3] and that parents/caregivers “*just seemed quite positive about it*” [ppt8] and “*were all pretty eager*” [ppt9]. Notably five participants commented that if they subsequently voluntarily implemented this intervention in their nurseries, they would not need to collect consent from parents to do this (see Additional File 3).

**Table 2:** The number of children (n) per nursery for whom consent was obtained from the eligible population, and the percentage of children recruited (%) per nursery

Nursery	Children consented (n)	Total children who could have been consented (n)	Percentage of children recruited (%)
Nursery A	10	14	71.4
Nursery B	24	29	82.8
Nursery C	57	109	52.3
Nursery D	26	35	74.3
Nursery E	60	201	29.9
Nursery F	42	50	84.0
Nursery G	7	7	100.0
Nursery H	59	124	47.6

### Acceptability of the Veggie Brek intervention and study processes to nursery staff

From the 6,066 cells where data should have been recorded on the data collection sheets, 3.9% (*n* = 238) were blank cells, meeting the green range of the progression criterion. The data also showed that the control and intervention group nurseries adhered to baseline and follow-up study processes with only 4.5% (*n* = 290/6384) of the cells showing missing data.

All staff (intervention group only) interviewed (*n* = 9) commented positively on the acceptability of the protocol, noting the ease of following the guidelines, remembering to offer the vegetables to the children, and completing the data collection sheets. One staff member commented that following the protocol “*was just really simple and easy*” [ppt3]. Regarding the feasibility of the protocol, eight interviewees reported that the protocol took some time to get used to but that the tasks soon became routine behaviour. Notably one staff member commented specifically that while the COVID-19 pandemic caused some disruption that it was still feasible to engage in all parts of the process: “*we managed it with skeleton staff during quite a big COVID time when we had lots of staff off, and we managed to do it*” [ppt8]. See Additional File 3 for more quotations from staff.

### Children's willingness to eat vegetables at breakfast during the Veggie Brek intervention

There were 1194 instances where staff indicated on the data collection sheet that a child was offered vegetables at breakfast time, 16 times (1.3%) where vegetables were not offered, and 16 times (1.3%) where staff did not provide any information under a child’s name. Where vegetables had been offered to a child at breakfast time, they ate some part of the vegetables (including both carrot and cucumber) 62.4% (*n* = 745/1194) of the time, meeting the green range of the progression criterion. Data for cucumber batons (only) showed that children ate some of the cucumber offered to them in 60.2% (*n* = 701/1165) of all instances. For carrot sticks (only), children ate some of the carrot offered to them in 34.7% (*n* = 391/1128) of all instances.

Eight nursery staff commented on children’s intrigue in the vegetables offered at breakfast which was characterised by questions being asked about why vegetables were being offered at breakfast and a willingness to try them. One participant commented that children were initially “*questioning ‘why have I got like carrot and cucumber sticks as my breakfast?’. We kind of expected that as it’s something new. They soon got used to have it, though. They was [sic] soon asking for it before we had already given it to them. So before we’d even given them cereal they was asking ‘are we having carrot and cucumber? Are we going to get it?’. After them having had it for a week or so, a couple of days, they were asking ‘can I have some?’ ‘can I have some carrot and cucumber for breakfast?*’” [ppt6]. All nine interviewees commented on the importance of repeated exposure for children to eat and like the vegetables (see Additional File 3). Indeed, one staff member commented on the positive effect repeated vegetable exposure had on children’s vegetable consumption throughout the day “*If we did offer a similar vegetable at snack time, they would tend to eat these generally better than they would have done. They were used to eating them before, not necessarily at breakfast time, at snack time, but they are having it more than once and getting used to the flavour and texture so would eat them more generally.”* [pp1].

Notably, five of the nine interviewees commented on children’s preference for the cucumber over carrots, with staff commenting that “*cucumber is easier for the younger children to eat*” [ppt5] and that “*cucumbers are soft, rather than that the carrots are quite hard*” [ppt3]. All interviewed staff also commented on children’s individual differences in how they interacted with the vegetables, either in regard to their acceptance of eating the vegetables, e.g., “*we had some children who would just eat all of them, and other children would be like ‘what on earth did you just give me?*’” [ppt3] or in regard to how the vegetables were eaten, such as in “*dipping it in their milk*” [ppt4] or in eating “*their normal breakfast, cereal or toast, and then move on to their veggies after*” [ppt9].

### Using photographs to record vegetable consumption

For 5.9% (*n* = 22/370) of photographs taken it was not possible to code the vegetables consumed (due to a blurred photograph, the vegetables being too distant in the photograph, the angle of the photograph not allowing for a clear sight of the vegetables, and/or the other vegetable pieces obstructing a clear view of other pieces). Eight of nine interviewees stated a preference for using the data collection sheets rather than using photographs. One staff member commented that “*for a busy working nursery, the sheets were easier*” [ppt9] with another stating that taking photographs was “*a little bit more stressful*” [ppt6]. Other reasons for preferring the data collection sheets were that staff perceived them to be more accurate for the researchers to understand how many vegetables had been eaten and that technological methods were not preferred.

## Discussion

The aim of this study was to assess the feasibility of the Veggie Brek trial methods and the acceptability of the Veggie Brek intervention where children were offered vegetables for breakfast alongside their main breakfast food. The recruitment of parents/caregivers to provide consent for their child(ren) to take part was mostly acceptable, meeting the amber stop–go criterion. Both acceptability of the intervention to nursery staff and children’s willingness to eat the vegetables met the green stop–go criteria. Overall, the intervention was implemented with good fidelity and was feasible and acceptable to nursery staff and children.

### Feasibility and acceptability of the Veggie Brek intervention

Although the recruitment data met the amber stop–go criterion, the average recruitment rate for children (68%) is higher than a comparable UK-based study (37%) where 12 nurseries (with 476 eligible children) were recruited to take part in a feasibility trial exploring a physical activity, nutrition and oral health intervention in nursery settings [33]. Furthermore, the recruitment of eight nurseries from the 12 contacted (67%) is also higher than the aforementioned study (31.6%). However, the number of children recruited varied between nurseries. It was higher in small-to-medium-sized nurseries (*n* = 7–50 children), which were in the amber or green range (72–100%), than in larger nurseries (*n* = 109–201 children), all of which were in the red range (30–52%). There was no evidence in the qualitative evaluation as to the reasons for lower recruitment in the larger nurseries. Indeed, the two example quotations (participants 8 and 9), noting that parents were positive about their child taking part, were from two (of the three) larger nurseries. It is possible that as larger nurseries have more complex organisational systems (e.g., more staff, nursery rooms, and children) collecting consent from parents/caregivers, either online or in person, may be more difficult to facilitate and may take longer. Also, as five nursery staff commented that they would feel it unnecessary to collect consent from parents if the nursery decided to routinely offer vegetables to children at breakfast, it is possible that staff felt unmotivated to collect consent from parents for this study. Together, these data suggest that, for a future definitive RCT, an opt-out consent process and a more extensive advertising campaign for parents/caregivers should be considered to optimise recruitment. Given the high number of children who took part in the study compared to the sample size recommended for feasibility trials [30], minor amendments to the recruitment protocol would allow for progression to a definitive trial.

The Veggie Brek intervention was highly acceptable to nursery staff. The quantitative data suggested that the intervention was implemented with high fidelity, with the qualitative data revealing that staff commented very positively about the intervention, both as a concept and practically. Staff felt that the intervention and evaluation processes could easily fit into their everyday morning routines with the activities quickly becoming routine. Staff also commented that they wanted to continue implementing the intervention after the end of the study.

Findings suggested that overall staff preferred the data collection sheets rather than taking food photographs. Photographs may allow for greater accuracy and precision of measuring children’s vegetable intake as the coding can be undertaken by a blinded independent assessor. However, the coding process revealed that photographs sometimes did not clearly show the leftover vegetables and, notably, the staff interview data revealed that data collection sheets were more feasible and acceptable to eight of the nine staff interviewed. This finding aligns with recent research which has indicated that logging food intake with photographs is viewed less favourably than using text [34]. Together, these data indicate that careful consideration needs to be given as to whether photographs are a viable approach to data collection in a subsequent trial.

Overall, children were willing to eat vegetables at breakfast time and nursery staff commented that children were interested in consuming them, frequently asking questions to staff about them. Interestingly, staff interviews revealed the manner in which children consumed the vegetables with some children treating the vegetables like a dessert (eating them after their main breakfast food) and others adding them to their main breakfast food. Staff also commented on the importance of repeated exposure – as has been highlighted previously in the literature [35] – and that later in the intervention some children started to ask for the vegetables before they were offered. Together, these data suggest that adult social norms pertaining to food-to-mealtime appropriateness were generally not evident for children in regard to vegetables at breakfast time in UK nursery/kindergarten settings. These data indicate that this intervention is acceptable to children of this age and that further research into offering children vegetables at breakfast time should be conducted to understand the benefits this may provide. That said, it is possible that the level of acceptability may be vegetable dependent, as data showed that many children preferred cucumber over carrots. For a future definitive RCT, substituting carrot for another vegetable (e.g., red peppers or sugar snap peas) should be considered. Future work should also explore the acceptability of highly rejected vegetables (e.g., broccoli) at breakfast time compared to other times in the day to further understand the role of breakfast time in facilitating healthy eating behaviours in childhood.

The findings of this study should be interpreted in the context of its strengths and limitations. In regard to strengths, it is the first study to investigate offering vegetables to children at breakfast time. It assessed different measures of collecting data on vegetable consumption (data collection sheets and photographs) to inform future research in this area. A large number of children were recruited to take part from nurseries of different sizes and set-ups, highlighting the pervasiveness of the intervention’s feasibility and acceptability across nursery types. This was a notable strength considering that the study was conducted during the COVID-19 pandemic, demonstrating the robustness of the intervention protocol. Finally, the mixed-methods approach facilitated a richer understanding of the intervention than using quantitative data alone, allowing for more-nuanced amendments to the intervention protocol before a future definitive RCT is undertaken. Regarding limitations, while our recruitment strategy sought nurseries of varying size and funding options for children’s attendance, the strategy did not ensure a diversity of nurseries in regard to relative deprivation. This should be remedied in a future definitive RCT by including a stratification based on nurseries’ index of multiple deprivation markers. Another limitation of the recruitment strategy is that the intended 2:1 cluster ratio was not achieved, as one (control group) nursery only collected consent from one child (of a possible 12) and was excluded. Notwithstanding the 3:1 ratio that this exclusion facilitated, the average demographic information for the children in the intervention and control group nurseries was similar. However, going forward into a definitive RCT, researchers should allocate more time and resource for the recruitment phase and include an internal pilot with a recruitment-based progression criterion to mitigate this risk.

The data also support the need to investigate the effectiveness of the Veggie Brek intervention in nurseries and kindergarten settings. From a public health perspective, an effectiveness trial is important to facilitate because UK children do not eat a sufficient amount of vegetables each day. The Veggie Brek intervention provides another time in the day where children are exposed to vegetables, with regular exposure a key factor in increasing the likelihood that children will like and eat vegetables [9]. Ultimately, an additional exposure will also likely lead to children consuming more vegetables each day, supporting optimal health, well-being and development across their lifespan. If a definitive trial were to demonstrate the intervention was effective, this would provide evidence to update food policies in nurseries as well as caregivers’ feeding guidelines/resources to promote vegetable consumption at breakfast in countries where this is not routinely implemented.

## Conclusion

In conclusion, offering vegetables to children at breakfast time in UK nursery/kindergarten settings was feasible to implement and acceptable to children and nursery staff. A definitive RCT should be undertaken, with minor modifications to the recruitment processes and data collection guidance for nursery staff, as the Veggie Brek intervention has the potential to positively impact the health of children.

## Supplementary Information

**Additional file 1. **Data collection sheet for the intervention phase.

**Additional file 2. **Schedule for semi-structured interview with nursery staff.

**Additional file 3. **Additional quotes from nursery staff provided in the interviews.

## Data Availability

The datasets used and/or analysed during the current study are available from the corresponding author on reasonable request. The trial protocol was preregistered on clinicaltrials.gov (ID: NCT05217550).

## Abbreviations

COVID-19: Coronavirus disease 2019
RCT: Randomised Controlled Trial
UK: United Kingdom
