# Supplementary figures and images for: Offering vegetables to children at breakfast time in nursery and kindergarten settings: the Veggie Brek feasibility and acceptability cluster randomised controlled trial
Source: Int J Behav Nutr Phys Act. 2023 Mar 28;20:38. doi: 10.1186/s12966-023-01443-z (PMC10043832; doi:10.1186/s12966-023-01443-z)

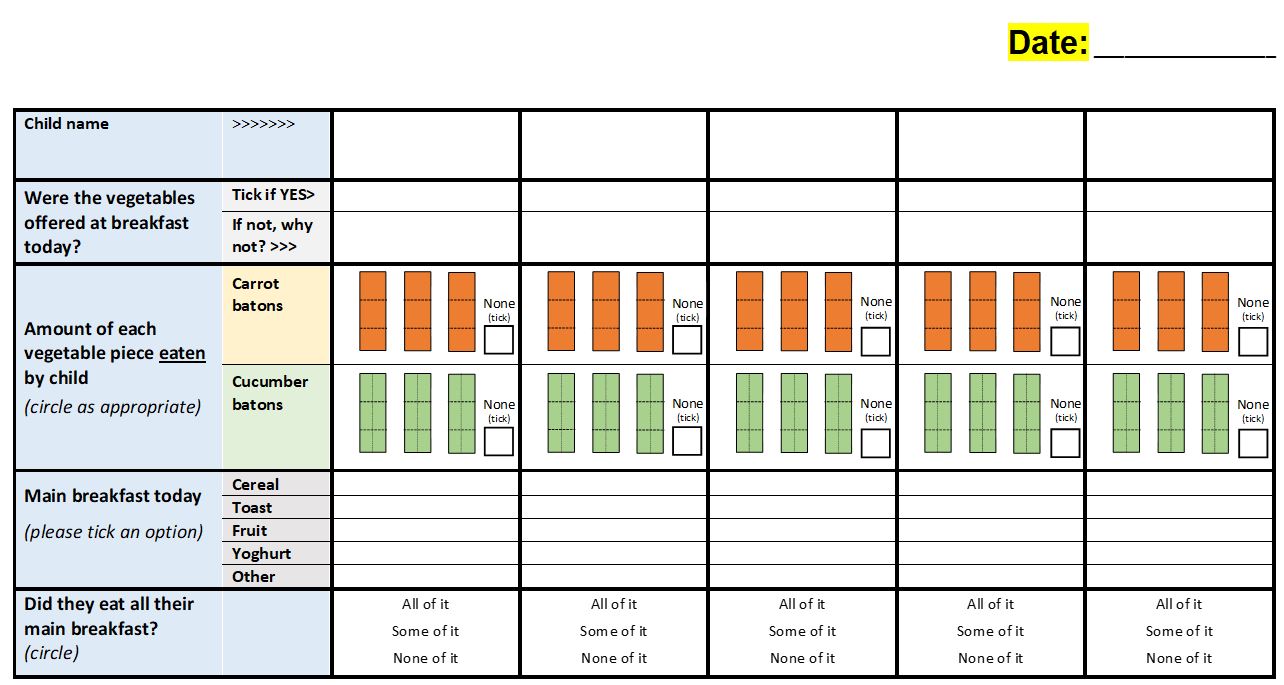

Supplement: Supplementary file 1 — Additional file 1. Data collection sheet for the intervention phase. [file 12966_2023_1443_MOESM1_ESM.jpg]
