# Supplementary material for: Offering vegetables to children at breakfast time in nursery and kindergarten settings: the Veggie Brek feasibility and acceptability cluster randomised controlled trial
Source: Int J Behav Nutr Phys Act. 2023 Mar 28;20:38. doi: 10.1186/s12966-023-01443-z (PMC10043832; doi:10.1186/s12966-023-01443-z)
Supplement: Supplementary file 2 — Additional file 2. Schedule for semi-structured interview with nursery staff. [file 12966_2023_1443_MOESM2_ESM.docx]

| 1 | How long have you worked in a nursery setting? |
| --- | --- |
| 2 | What were your initial thoughts when you heard about the Veggie Brek study? |
| 3 | Would you have considered offering vegetables to children at breakfast time prior to taking part in this research? |
| 4 | In general, can you describe the children’s experience of being offered vegetables at breakfast time at the nursery? (prompt: do you believe they liked eating the vegetables? Did they interact with the vegetables at all? Were they ever distracted (from eating their usual breakfast) by the vegetables?) |
| 5 | What were the children’s conversations like surrounding being offered vegetables at breakfast time? |
| 6 | How did you find taking part in the study? |
| 7 | Did you find it easy to follow the guidelines for what you were required to do in this study? (prompts: was it easy to remember to serve the vegetables? how did you find taking photographs of the children’s leftovers compared to completing the data collection sheet?) |
| 8 | Is there anything that you’d suggest should be changed about the way the study was run if we were to do it again? |
| 9 | Do you believe that offering vegetables at breakfast is a valuable way to help children to eat more veg? |
| 10 | What do you think other nursery staff would say about this research? |
| 11 | Is there anything else that you’d like to comment on about this study, either to improve it or just in general? |
