# Supplementary material for: Offering vegetables to children at breakfast time in nursery and kindergarten settings: the Veggie Brek feasibility and acceptability cluster randomised controlled trial
Source: Int J Behav Nutr Phys Act. 2023 Mar 28;20:38. doi: 10.1186/s12966-023-01443-z (PMC10043832; doi:10.1186/s12966-023-01443-z)
Supplement: Supplementary file 3 — Additional file 3. Additional quotes from nursery staff provided in the interviews. [file 12966_2023_1443_MOESM3_ESM.docx]

| Outcome | Participant number | Quote |
| --- | --- | --- |
| Recruitment  [“Would you have to collect consent from parents to routinely introduce vegetables at breakfast time?] | 1 | “Um, we could have just done it, but I think initially we would have to be confident in giving the parents feedback, and we haven’t really done that on this study because nobody really asked. I think if they knew their child was enjoying it and getting a lot out of it, we would be willing to do that.” |
|  | 3 | “No, if we want to, we can feed them anything we want pretty much. So yes, we give them a list of what we feed them and we’ll just put it on the app and just say this is what they’ve eaten so we don’t actually have to ask them for permission to do it.” |
|  | 4 | “As we said, we don’t take the consent as part of the policy.” |
|  | 5 | “No, I could just do that.” |
|  | 6 | “No, it was just something we would change and pass on to the parents during pick-up time.” |
| Acceptability of the intervention | 5 | “It was all very well structured and easy for us to do” |
|  | 2 | “I think the first day most probably was a little bit stressful, getting it all ready, but then afterwards it was fine” |
|  | 8 | “After the first few days, it just became like second nature” |
|  | 5 | “It became quite routine … [after] 2-3 weeks it felt kind of normal” |
|  | 8 | “A positive experience” |
|  | 7 | “I think a lot of nurseries should implement it into their breakfast routine.” |
|  | 8 | “[after the study finished] we did yellow peppers for snack and breakfast time and they all got eaten – we just put them on a plate in the middle of the table and they went down a storm.” |
|  | 8 | “It has made an impact on our breakfasts because we have started to offer veggies – not every day, but now and then when we do have them in the fridge we’ll go “oh, we’ll have some pepper with breakfast today” and it has – yeah, changed our – put less of a weird thing. We’ve just gone “oh, we’ll have some carrot today, or some cucumber, or some peppers”. So, yeah, very positive.” |
|  | 9 | “I thought [taking part in the study] was OK. Obviously having it all set up – and we did it quite particularly and things – so that for a busier day was a little bit tricky, but I think in terms of if we started doing it full time, I don’t think it would have to be as particular so it would be easier.” |
|  | 3 | “yeah I really love the idea. I thought it was a really good idea.” |
|  | 3 | “I think if vegetables are offered more throughout the day, they’re more likely to eat them because they’re more – what’s the word – in contact with them? So they’re more – yeah – I think it’s a really good idea for breakfast.” |
|  | 4 | “I feel like we should do it more like this, rather than not for the six weeks, but you putting it in our practice and actually we make sure that we are going to introduce that to children every day now from the morning, and I imagine myself and other colleagues will speak to management as well.” |
|  | 6 | “I think it was a great idea giving them the vegetables at breakfast” |
| Children's willingness to eat vegetables at breakfast | 1 | “To begin with it was quite slow, the children were getting used to it, but as they got used to it, they began to eat it a bit more and were asking for it daily. Some children were often asking for seconds and things. I think it’s definitely changed our perception of what children could be offered at breakfast; you know.” |
|  | 1 | “Never really thought about vegetables being part of a typical breakfast meal. Now we’ve realised that children did take to it well, and it could be part of a future offering.” |
|  | 1 | “There were a couple of children that would say “more cucumber” or something like that.” |
|  | 2 | “[the children] were really happy to actually try it.” |
|  | 2 | “One child I remember for the first four days I said to him, would you like some … and he was like, no, no, no … and I think on the fifth, day, he actually had them.” |
|  | 3 | “Yeah, most of them ate like a spoonful of their breakfast and then ate a carrot stick or and then another one. But some children ate all their breakfast and then went on as if it was kind of a pudding afterwards. Because what we do normally is after lunchtimes we have our main meal and then we have a fruit afterwards, so I think they’ve just got a routine for them.” |
|  | 4 | “They like playing with and like dipping it in their milk, trying to not eat the skins, just eat the middle, soft bit. Some children tried to put them in the cereals, and like exploring them with the food.” |
|  | 7 | “At first it was a bit like ‘carrot, breakfast, don’t want to eat it’, but then obviously once they got into it, doing it, over the course of the week, they knew that it was there, and the children liked – the majority of the children like carrot and cucumber anyway. And it just came naturally that they had their Weetabix for example, they had their fruit, and then it was ‘I’ve got my vegetables, I’m going to eat them before I get down from the table’” |
|  | 9 | “Over time a lot more of them would eat them, or nibble them at least, or give them a play with. Some of them started dipping them in their cereal, and then you had some children who were not phased at all and looked at them and were like ‘yes’ and ate their entire box, and it was never an issue, and they didn’t have to kind of get used to the routine. It was like from the get-go they kind of just ate it all, and that was how it went.” |
|  | 9 | “There was a couple that – there was a couple of children that are like super fussy with veg and won’t go near it. And they seemed a little bit more open to it in the meal times, and were more easily encouraged to try them. Because you know they’d been encouraged to eat their veggies and breakfast and things, so it definitely had an impact in that kind of sense. It wasn’t a huge one, but you know the more they saw the veg, it was offered to them, it obviously then resonated throughout the rest of the day and they weren’t like appalled by being given veg at lunchtime.” |
